# Comparison of the incidence of recovery agitation with two different doses of ketamine in procedural sedation: A randomized clinical trial

**DOI:** 10.1111/acem.15116

**Published:** 2025-01-29

**Authors:** Çağrı Türkücü, İsmet Parlak, Kamil Kokulu, Ekrem T. Sert, Hüseyin Mutlu

**Affiliations:** ^1^ Department of Emergency Medicine Aksaray Training and Research Hospital Aksaray Turkey; ^2^ Department of Emergency Medicine Aksaray University School of Medicine Aksaray Turkey

**Keywords:** emergency department, ketamine, procedural sedation, recovery agitation

## Abstract

**Objectives:**

The objective was to compare the incidence of recovery agitation and efficacy of two different intravenous (IV) doses of ketamine (0.5 mg/kg vs. 1 mg/kg) in adult patients who presented to the emergency department (ED) requiring procedural sedation with ketamine.

**Methods:**

This randomized, prospective clinical trial included patients aged 18–75 years who required procedural sedation with ketamine in the ED. Patients were randomized to receive IV ketamine at either 0.5 mg/kg (low dose) or 1 mg/kg (high dose). The primary outcome was the incidence of recovery agitation, assessed by the Richmond Agitation–Sedation Scale (RASS) at 5, 15, and 30 min following the procedure, in both dosage groups. Secondary outcomes included overall efficacy, sedation duration, and changes in vital signs.

**Results:**

A total of 108 patients were enrolled in the study, 54 in each group. The median (IQR) RASS scores at 5, 15, and 30 min were −4 (−5 to −4), −1 (−1.3 to 0), and 0 (−1 to 0.5), respectively, in the low‐dose group and −4 (−5 to −4), −1 (−3 to 0), and 0 (0 to 0), respectively, in the high‐dose group. The incidence of recovery agitation was similar between the low‐ and high‐dose groups (difference 1.9%, 95% confidence interval [CI] −14.8% to 18.4%). No significant difference was observed in sedation duration between the two groups (difference 0%, 95% CI −3.0% to 4.0%). While no additional ketamine was required in the high‐dose group, four patients (7.4%) in the low‐dose group required an additional half‐dose (difference 7.4%, 95% CI −2.3% to 18.7%). Changes in vital signs were similar between the two groups.

**Conclusions:**

There was no significant difference in recovery agitation, sedation duration, and changes in vital signs between 0.5 and 1 mg/kg IV ketamine for procedural sedation in the ED.

## INTRODUCTION

Patients presenting to the emergency department (ED) frequently require procedural sedation and analgesia for painful procedures. Ketamine is a widely used and effective sedative‐analgesic, particularly in pediatric procedural sedation and analgesia.^1^, ^2^ However, the use of ketamine in adult ED patients may be limited due to concerns about recovery agitation, also known as the emergence phenomenon or dysphoric reaction.

A study by Green et al.^3^ evaluating 8282 pediatric patients who received ketamine for painful procedures found a correlation between higher intravenous (IV) doses and an increased incidence of recovery agitation. Although recovery agitation is reported to occur more frequently in adults than in children,^2^ the relationship between ketamine dosage and recovery agitation in adults remains unclear. Previous studies have compared the incidence of recovery agitation with combinations of IV ketamine and other sedatives, such as propofol,^4^ midazolam,^5^ and haloperidol.^6^ Despite recommendations for IV ketamine doses ranging from 0.5 to 2 mg/kg for procedural sedation in adults,^2^, ^5^, ^7^, ^8^, ^9^, ^10^, ^11^ to the best of our knowledge, there are no known randomized trials comparing different IV ketamine doses in terms of recovery agitation incidence.

If the incidence of recovery agitation differs between low and high doses of IV ketamine in adults, ED physicians may opt to adjust initial dosing strategies to minimize this side effect. The primary objective of this study was to compare the incidence of recovery agitation between adults receiving two different doses of IV ketamine (0.5 mg/kg vs. 1 mg/kg). Secondary objectives included the comparison of the efficacy of these doses, sedation duration, and changes in vital signs.

## MATERIALS AND METHODS

### Study design and setting

This prospective, randomized, single‐blind clinical trial was conducted at the ED of a tertiary training and research hospital between January 2023 and September 2023. The study received approval from the institutional ethics committee and was registered on ClinicalTrials.gov (NCT05786365). Written informed consent was obtained from all patients prior to participation. The study was conducted and reported according to the Consolidated Standards of Reporting Trials (CONSORT) guidelines.^12^

### Selection of participants

Patients who were determined by the treating attending emergency physician to require procedural sedation and analgesia were evaluated for eligibility by the study investigators. Adult patients aged 18–75 were enrolled. The exclusion criteria were pregnancy, a history of liver or kidney transplantation, liver failure, renal dysfunction, refusal to participate, altered mental status, active coronary artery disease, conditions affecting consciousness such as substance or alcohol use, chronic obstructive pulmonary disease, patients with active unstable medical or trauma conditions, and heart failure.

### Study protocol and interventions

The participants were randomized into two groups using a block randomization algorithm: a low‐dose group (0.5 mg/kg ketamine) and a high‐dose group (1 mg/kg ketamine). The randomization schedule was prepared with random allocation software and recorded on the computer in the procedural sedation area of the ED. Each new subject was assigned to the next number according to the randomization schedule after enrolled by the study investigators. Only patients were blinded to the administered agent. Before procedural sedation and analgesia (immediately prior to IV ketamine administration), each patient's vital signs (blood pressure, heart rate, respiratory rate, temperature, and oxygen saturation) were measured and recorded. During sedation, all patients were continuously monitored via a three‐lead electrocardiogram; heart rate, respiratory rate, and peripheral oxygen saturation were measured. Blood pressure was recorded every 5 min using an automatic blood pressure monitor.

For both groups, ketamine (500 mg/10 mL vial of ketamine hydrochloride, Pfizer, Turkey) was diluted in a 1:1 ratio with 0.9% saline and administered as an IV push over 1 min prior to the painful procedure. Sedation level was evaluated using the Richmond Agitation–Sedation Scale (RASS). Procedures were initiated once patients achieved a minimum RASS score of −4 (indicating deep sedation). If sufficient sedation (RASS score of −4) was not achieved within the first 5 min following ketamine administration, a half‐dose of IV ketamine was given again. Patients who failed to reach a RASS score of −4 after this additional dose were excluded from the study.

### Data collection and processing

Demographic data, vital signs, procedure type, RASS scores, sedation duration, additional dose requirements, and the presence of adverse effects were recorded using standardized data collection forms. Emergency physician investigators assessed and recorded RASS scores at 5, 15, and 30 min following ketamine administration. Painful procedures (e.g., laceration repair) were performed by an emergency physician other than the study investigators. The RASS ranges from −5 to +4, with 0 indicating an alert and calm state. Scores from −1 to −5 reflect increasing levels of sedation, while scores from +1 to +4 indicate increasing levels of agitation and combative behavior.^13^ Recovery agitation was defined as a RASS score of +2 or higher following ketamine administration. Patients with a RASS score of +4, indicating overtly combative or violent behavior, were considered to have clinically important recovery agitation.

Sedation duration was defined as the time from initial IV ketamine administration to the point when the patient's RASS score returned to 0. We determined sedation efficacy according to whether additional ketamine doses were needed to reach RASS score of −4 points. Respiratory adverse effects included laryngospasm (stridor or other evidence of airway obstruction that did not improve with airway alignment maneuvers),^3^ desaturation (oxygen saturation <90%), and apnea (a minimum 20‐s transient cessation of breathing). Postketamine hypertension was defined as a systolic blood pressure exceeding 140 mm Hg or an increase of more than 20% from baseline systolic blood pressure. Hypotension was defined as a systolic blood pressure <100 mm Hg. Patients who experienced vomiting from the time of IV ketamine administration until discharge were documented on the study form as “vomiting present.” Following the recovery phase, each patient was asked whether they experienced nausea. If the patient responded “yes,” it was recorded on the study form as “nausea present,” regardless of severity. Patients who experienced vomiting were not asked about nausea.

### Outcome measures

The primary outcome was the incidence of recovery agitation, as evaluated by RASS scores at 5, 15, and 30 min after the procedure in both the low‐dose and the high‐dose ketamine groups. Secondary outcomes included the efficacy of the two ketamine doses and the frequency of adverse effects unrelated to recovery agitation.

### Statistical analysis

Based on a previous study,^5^ it was assumed that the incidence of recovery agitation in the high‐dose ketamine group (1 mg/kg) would be 25%. To detect a 20% difference between the two groups, a chi‐square test with a two‐sided hypothesis, minimum power of 80%, and an alpha value of 5% indicated that a sample size of at least 49 patients per group (98 total) would be required. Accounting for a 10% dropout rate, we aimed to enroll 108 patients. Statistical analysis was performed using SPSS software (Version 20.0, SPSS Inc.), with *p*‐values <0.05 considered statistically significant. The Kolmogorov–Smirnov test was used to assess the normality of the data distribution. Continuous variables were expressed as mean ± standard deviation or median and interquartile range (IQR) values, depending on their distribution. Categorical variables were reported as numbers and frequencies. Group comparisons for categorical variables were performed using the chi‐square test or Fisher's exact test, while Student's *t*‐test was used for normally distributed continuous variables and the Mann–Whitney *U*‐test for nonparametric data.

## RESULTS

**FIGURE 1:**
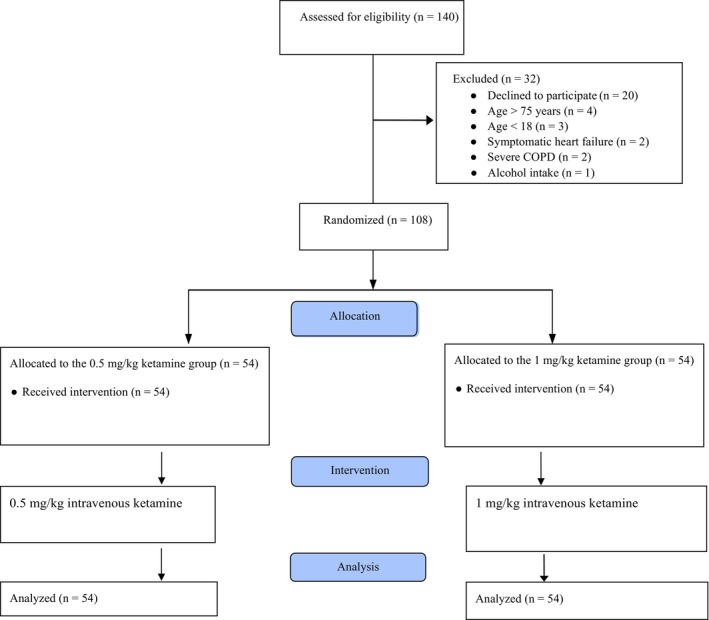
CONSORT flow diagram of the study.

Of the 140 patients assessed for eligibility, 108 were enrolled in the study, with 54 in the low‐dose group and 54 in the high‐dose group (Figure 1). The median (IQR) age of the participants was 48 (32–70) years, and 63% of the patients (*n* = 68) were male. There were no significant differences between the groups in terms of baseline characteristics and demographics (Table 1).

**TABLE 1:** Baseline characteristics of the participants.

Characteristic	Ketamine groups		Difference (95% CI)
	Low dose (0.5 mg/kg)	High dose (1 mg/kg)	
	*n* = 54	*n* = 54	
Age (years)	49.5 (32.8–70.0)	46.5 (30.8–72.0)	0.7 (−5.0 to 6.0)
Gender, male	32 (59.3)	36 (66.7)	7.4 (−11.8 to 25.9)
Weight (kg)	80.0 ± 14.0	77.5 ± 14.2	
Procedure			
Fracture/dislocation reduction	19 (35.3)	18 (33.3)	1.9 (−16.9 to 20.4)
Abscess incision	13 (24.1)	12 (22.2)	1.9 (−15.2 to 18.8)
Central venous catheter placement	11 (20.4)	7 (13.0)	7.4 (−8.2 to 22.7)
Chest tube placement	4 (7.4)	5 (9.3)	1.9 (−10.9 to 14.7)
Other	7 (13.0)	12 (22.2)	9.3 (−6.6 to 24.7)
Baseline vital signs			
Heart rate (beats/min)	89.2 ± 16.8	92.3 ± 14.2	−3.1 (−9.0 to 2.9)
Systolic blood pressure (mm Hg)	128.3 ± 14.9	130.2 ± 12.4	−1.9 (−7.2 to 3.3)
Diastolic blood pressure (mm Hg)	79.4 ± 9.5	79.8 ± 10.4	−0.4 (−4.2 to 3.4)
O_2_ saturation (%)	96.6 ± 2.5	97.1 ± 2.2	−0.5 (−1.4 to 0.4)

*Note*: Categorical variables are expressed as *n* (%), and continuous variables are expressed as mean (±SD) or median (IQR), as appropriate.

Table 2 presents the RASS scores obtained at 5, 15, and 30 min for both groups. The incidence of recovery agitation (RASS ≥+2) was 20.4% in the low‐dose group and 22.2% in the high‐dose group, but this difference was not statistically significant (difference  1.9%, 95% confidence interval [CI] −14.8% to 18.4%). Clinically important recovery agitation (RASS 4) occurred in one patient in the low‐dose group. Sedation duration was similar between the low‐dose (21.5 [17–30] min) and high‐dose (20 [16.8–27] min) groups (difference 0%, 95% CI −3.0% to 4.0%). While no patients in the high‐dose group required additional ketamine to achieve adequate sedation (RASS ≤−4), four patients (7.4%) in the low‐dose group required a half‐dose supplemental ketamine, although this difference was not statistically significant (difference 7.4%, 95% CI −2.3% to 18.7%).

**TABLE 2:** Summary of outcomes and differences between groups.

Variables	Ketamine groups		Difference (95% CI)
	Low dose (0.5 mg/kg)	High dose (1 mg/kg)	
	*n* = 54	*n* = 54	
RASS scores			
At 5 min	−4 (−5 to −4)	−4 (−5 to −4)	0 (0 to 0)
At 15 min	−1 (−1.3 to 0)	−1 (−3 to 0)	0 (0 to 1)
At 30 min	0 (−1 to 0.5)	0 (0 to 0)	0 (0 to 0)
Recovery agitation incidence^a^	11 (20.4)	12 (22.2)	1.9 (−14.8 to 18.4)
RASS at 5 min			
+1 (restless)	2 (3.7)	0 (0)	3.7 (−5.1 to 13.8)
+2 (agitated)	2 (3.7)	2 (3.7)	0 (−10.6 to 10.6)
+3 (very agitated)	0 (0)	0 (0)	0 (NA)
+4 (combative)	0 (0)	0 (0)	0 (NA)
RASS at 15 min			
+1 (restless)	1 (1.9)	2 (3.7)	1.9 (−8.0 to 12.1)
+2 (agitated)	6 (11.1)	9 (16.7)	5.6 (−9.2 to 20.2)
+3 (very agitated)	0 (0)	0 (0)	0 (NA)
+4 (combative)	1 (1.9)	0 (0)	1.9 (−6.6 to 11.1)
RASS at 30 min			
+1 (restless)	3 (5.6)	1 (1.9)	3.7 (−6.5 to 14.6)
+2 (agitated)	2 (3.7)	1 (1.9)	1.9 (−8.0 to 12.1)
+3 (very agitated)	0 (0)	0 (0)	0 (NA)
+4 (combative)	0 (0)	0 (0)	0 (NA)
Sedation time (min)	21.5 (17.0–30.0)	20.0 (16.8–27)	0 (−3.0 to 4.0)
Patients requiring additional ketamine	4 (7.4)	0 (0)	7.4 (−2.3 to 18.7)

*Note*: Data are reported as median (IQR) or *n* (%).

Abbreviation: RASS, Richmond Agitation–Sedation Scale.

^a^ Recovery agitation incidence was defined as the proportion of patients with a RASS score of ≥+2.

Secondary outcomes are shown in Table 3. The incidence of transient hypertension and tachycardia was similar between the low‐ and high‐dose ketamine groups. No significant differences were observed between the groups regarding respiratory adverse effects. One patient in the low‐dose group experienced oxygen desaturation, which was promptly resolved with supplemental oxygen and repositioning of the airway. Gastrointestinal side effects (nausea or vomiting) were observed in 10.2% (*n* = 11) of all patients, with no significant difference between the groups. One patient (in the low‐dose group) with transient hypotension was treated with a 500 mL IV saline bolus.

**TABLE 3:** Secondary outcomes in the study groups.

Adverse effects	Ketamine groups, *n* (%)		Difference (95% CI)
	Low dose (0.5 mg/kg)	High dose (1 mg/kg)	
	*n* = 54	*n* = 54	
Tachycardia	5 (9.3)	4 (7.4)	1.9 (−10.9 to 14.7)
Hypertension	20 (37.0)	23 (42.6)	5.6 (−13.8 to 24.4)
Hypotension	1 (1.9)	0 (0)	1.9 (−6.6 to 11.1)
Laryngospasm	0 (0)	0 (0)	0 (NA)
Oxygen desaturation	1 (1.9)	0 (0)	1.9 (−6.6 to 11.1)
Apnea	0 (0)	0 (0)	0 (NA)
Nausea or vomiting	5 (9.3)	6 (11.1)	1.9 (−11.6 to 15.4)
Diplopia/nystagmus	10 (18.5)	10 (18.5)	0 (−16.0 to 16.0)
Clinically important recovery agitation	1 (1.9)	0 (0)	1.9 (−6.6 to 11.1)

## DISCUSSION

In this prospective randomized clinical trial, we found no significant difference in the incidence of recovery agitation between high‐dose (1 mg/kg) and low‐dose (0.5 mg/kg) IV ketamine in patients undergoing procedural sedation and analgesia in the ED. Furthermore, there was no significant difference in efficacy between the two ketamine doses. Given that ketamine does not exhibit a clear dose–response continuity specific to titration during procedural sedation, we consider that our results are clinically relevant.

In this study, the incidence of ketamine‐induced recovery agitation was 20.4% in the low‐dose group and 22.2% in the high‐dose group, with clinically important agitation occurring in only 0.9% of cases. Previous studies have reported recovery agitation incidences ranging from 0% to 64% in adult patients following ketamine sedation across the United States and Europe.^6^, ^7^, ^8^, ^14^, ^15^, ^16^ The wide range of incidence rates could be attributed to social, cultural, environmental, or genetic factors. Furthermore, this variability could stem from differences in the definition, classification, and measurement of recovery agitation, as some studies did not specify the symptoms constituting recovery agitation or how it was defined.^7^, ^14^ While some authors defined recovery agitation based on patient experiences such as nightmares or unpleasant dreams,^5^, ^16^ others based it on physician observations during sedation.^10^, ^17^ However, none of these methods are entirely objective.

In our study, we used the RASS, a reliable and internationally validated scale with high inter‐rater reliability, to detect recovery agitation.^18^, ^19^ We set the threshold for recovery agitation at RASS ≥+2, in light of previous studies.^20^, ^21^ We did not consider patients with RASS scores of +1 (restless) as having recovery agitation, as this could be influenced by the patient's clinical/psychological state or existing pain (e.g., dislocated shoulder). Some studies, however, have used a threshold of RASS ≥+1 to define recovery agitation, reporting incidences as high as 60%.^19^, ^22^

In a study conducted by Newton and Fitton,^7^ 0.5 mg/kg IV ketamine was administered to 46 adult patients admitted to the ED for procedural sedation, and a sufficient level of sedation was achieved in all patients to allow the procedure to be completed without the need for additional doses of ketamine. In contrast, four of the 54 patients in the low‐dose group of our study required supplemental half‐doses of ketamine to reach the target sedation level.

## LIMITATIONS

This study has several limitations. First, the single‐center design limits the generalizability of our findings. Second, we included only adult patients aged 18 to 75 years; therefore, our results may not apply to other age groups. Lastly, the lack of a universally accepted definition for recovery agitation and the use of different rating scales to measure agitation across studies prevented the direct comparison of our results with previous research.

## CONCLUSIONS

We found no difference in recovery agitation, efficacy, duration of sedation, or changes in vital signs between 0.5 and 1 mg/kg IV ketamine for procedural sedation and analgesia in the ED. Based on our findings, 0.5 mg/kg IV ketamine may be considered a suitable starting dose for procedural sedation and analgesia, avoiding the need for higher doses.

## CONFLICT OF INTEREST STATEMENT

No conflict of interest was declared by the authors.

## Data Availability

The data that support the findings of this study are available from the corresponding author upon reasonable request.
